# Phosphoglycerate mutase 2 is elevated in serum of patients with heart failure and correlates with the disease severity and patient’s prognosis

**DOI:** 10.1515/med-2021-0324

**Published:** 2021-08-11

**Authors:** Min Li, Xiaoyuan Gao, Huiyun Wang, Mingli Zhang, Xiaoying Li, Shuya Wang, Shaoqin Wang, Chongfeng Cao, Ying Li, Guohai Su

**Affiliations:** Department of Emergency, Jinan Central Hospital, Cheeloo College of Medicine, Shandong University, Jinan 250100, China; Research Center of Translational Medicine, Jinan Central Hospital, Cheeloo College of Medicine, Shandong University, Jinan 250100, China; Department of Cardiovascular Medicine, Jinan Central Hospital, Cheeloo College of Medicine, Shandong University, Jinan 250100, China; Research Center of Translational Medicine, Jinan Central Hospital, Cheeloo College of Medicine, Shandong University, No. 105 Jiefang Road, Lixia District, Shandong Province, Jinan 250100, China; Department of Intensive Care Unit, Jinan Central Hospital, Cheeloo College of Medicine, Shandong University, Jinan 250100, China; Department of Cardiovascular Medicine, Jinan Central Hospital, Cheeloo College of Medicine, Shandong University, No. 105 Jiefang Road, Lixia District, Shandong Province, Jinan 250100, China

**Keywords:** HF, biomarker, PGAM2, NYHA, cardiac function grading

## Abstract

**Background:**

Heart failure (HF) is a serious and advanced stage of various cardiac diseases with high mortality and rehospitalization rates. Phosphoglycerate mutase 2 (PGAM2) overexpression was identified in the serum of patients with HF.

**Material/methods:**

One hundred and fifty-three cases of HF were included in the present work. According to New York Heart Association (NYHA) classification, 22 were grade II, 84 were grade III, and 47 were grade IV. Serum PGAM2, NT-proBNP, B-type natriuretic peptide (BNP), troponin T (TNT), and Cys-C of HF patients were detected using ELISA assay. Left ventricular ejection fraction, left ventricular end-diastolic inner diameter, and left atrium (LA) inner diameter of the included cases were also detected by the cardiac color Doppler.

**Results:**

The number of patients with atrial fibrillation was significantly higher in NYHA IV group than in groups II and III with statistical difference (*p* < 0.05). The serum PGAM2, NT-proBNP, and Cys-C were significantly higher in NYHA IV group than in NYHA II and NYHA III groups (*p*_all_ < 0.05). NT-proBNP had the highest prediction efficacy of HF severity and PGAM2 was also a potential biomarker for HF severity evaluation with relatively high sensitivity, specificity, and area under the ROC. The overall survival among NYHA II, III, and IV groups were statistically different (*p* = 0.04) with the median survival time of 25 months for NYHA III and IV groups.

**Conclusion:**

PGAM2 is a new promising biomarker for evaluation of the severity of HF. Combination detection using multiple serum factors such as PGAM2, NT-proBNP, BNP, TNT, and Cys-C can improve the HF severity differential diagnosis performance.

## 1 Introduction

Heart failure (HF) is a disease with poor prognosis and frequent occurrence [1,2,3]. The severity and prognosis of various biomarkers for HF have been widely discussed, including B-type natriuretic peptide (BNP), NT-proBNP [4,5,6], neutrophils gelatinase-related lipid calin [7], soluble ST2, troponin, central preadrenal medlin, copeptin, chromogranin A, and S100B protein [8]. BNP is considered as an important diagnostic marker of HF [9]. Although the accuracy of BNP in diagnosing chronic HF and acute decompensated HF has been widely assessed and specific boundaries established, the BNP test is subject to limitations that may challenge its interpretation. These included values in the middle gray area and explained subtle differences in levels of renal dysfunction, obesity, and advanced age.

Phosphoglycerate mutase (PGAM) is an important enzyme in glycolysis and gluconeogenesis pathways, which can catalyze the mutual transformation between 3-DPGA and 2-DPGA, and is widely found in various tissues [10]. Studies have shown that PGAM can be activated under oxidative stress and participates in regulating tumor cell proliferation, indicating that PGAM not only has catalytic function but also has important regulatory function [10]. The application of biochemical automatic analyzer to determine the PGAM significantly increased in acute myocardial infarction (AMI) and cerebral hemorrhage. PGAM2 is mainly expressed in skeletal muscle and myocardium. Previous study indicated that constitutive upregulated PGAM2 affects stress resistance of heart in mice [11]. However, little study has been performed on PGAM2 in cardiac patients, especially those with HF. In a previous study, PGAM2 secretion was found to increase in the outflow after ischemia of donor hearts *in vitro* [12]. In another study, in a mouse model of transverse aortic constriction, change in glycolysis and tricarboxylic acid circulation in mice overexpressed PGAM2-induced impaired myocardial systolic function and decreased heart tolerance to stress load earlier.

In the present work, we aim to analyze PGAM2 expression in HF cases, and evaluate its correlation with New York Heart Association (NYHA) classification, which could be used as a serological marker for HF severity evaluation.

## 2 Methods

### 2.1 Patients

**Figure 1:**
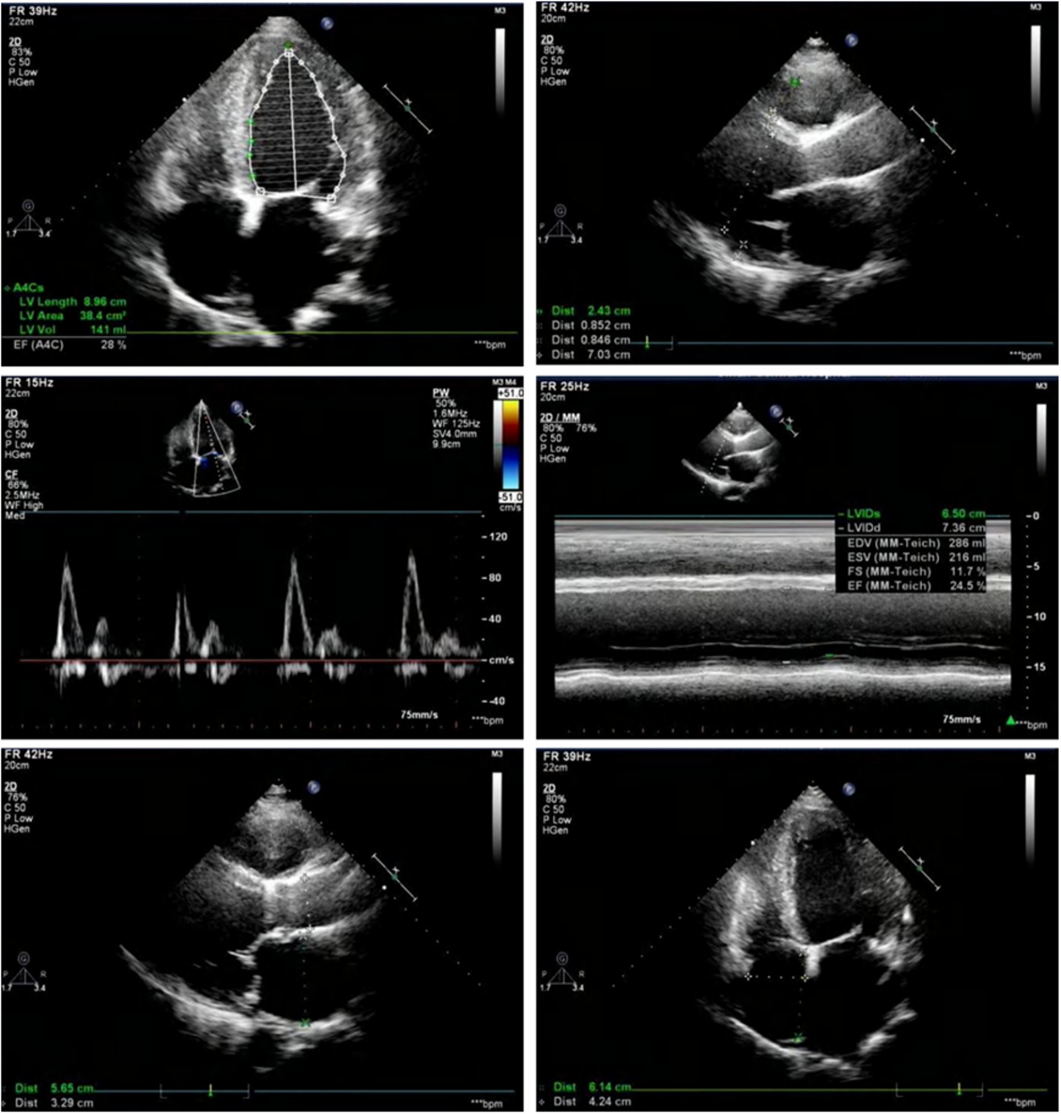
Echocardiography examination of HF cases.

Patients were enrolled from October 2016 to February 2018. The patients’ inclusion criteria were as follows: (1) All the patients were diagnosed with HF according to the 2014 guidelines for diagnosis and treatment of HF [13]; (2) Age should be more than 30 years. The patients’ exclusion criteria were as follows: (1) complicated with malignant tumor diseases; (2) serious dysfunction of liver, kidney, and other important organs; (3) immune diseases or systemic infectious diseases; (4) pregnant or lactating women. Twenty healthy people were selected as control group. Color Doppler ultrasound was used to measure the patients’ left ventricular ejection fraction (LVEF), left ventricular end-diastolic inner diameter (LVEDD), left atrium (LA) inner diameter, and other indicators, as shown in Figure 1. Twenty healthy people were selected as control group. This work was approved by the ethical committee of Jinan Central Hospital, Cheeloo College of Medicine, Shandong University. Written informed consents were obtained from all the included subjects.

### 2.2 Blood sampling test of BNP and other indicators

Venous blood specimens were collected on the morning of the second day after admission or on the day of physical examination and sent to the biochemistry room of Jinan Central Hospital for examination and serum level of PGAM2, T-proBNP, BNP, troponin T (TNT), and Cys-C were detected.

### 2.3 Serum PGAM2 detection by ELISA assay

On the morning of the second day after admission or on the day of physical examination, 3 mL of elbow venous blood was extracted under fasting state, and centrifuged at 3,500 rpm for 10 min. ELISA assay was used to detect serum PGAM2. The instrument used was Model 450 enzyme marker (Bio-rad, USA). The kit was provided by Beijing Equation Biotechnology Co., Ltd. The specific operation was conducted in strict accordance with the kit instructions

### 2.4 Statistical analysis

SPSS 18.0 statistical software was used in this study. Measurement data were expressed as \bar{x}\pm s], independent sample *t* test and one-way analysis of variance were used for intergroup comparisons, and student-*t* test was used for pairwise comparison between multiple groups. *p* < 0.05 was considered statistically significant. Correlation analysis of serum PGAM2 and other indicators was conducted by Pearson, and the clinical value of serum PGAM2 was tested by ROC curve.

## 3 Results

### 3.1 General characteristic of the included HF cases

The general baseline characteristics of the three groups are demonstrated in Table 1. There were no statistical difference in the aspects of gender, age, AMI, diabetes mellitus, coronary heart disease, stroke history, renal insufficiency, and hypertension (*p*_all_ > 0.05). However, the patients with atrial fibrillation in group IV were significantly higher than in groups II and III with statistical difference.

**Table 1:** The main characteristics of the included cases

Characteristic	II (*n* = 22)	III (*n* = 84)	IV (*n* = 47)	*F*/chi-square	*p*-value
Age	74.50 ± 10.71	77.32 ± 9.84	78.91 ± 12.24	1.27	0.28
Gender (*n*, [%])				0.81	0.67
Male	10	49	27		
Female	12	35	20		
AMI				1.25	0.53
Positive	2	3	3		
Negative	20	81	44		
Diabetes mellitus				0.25	0.88
Positive	7	27	17		
Negative	15	57	30		
Coronary heart disease				0.13	0.94
Positive	7	26	16		
Negative	15	58	31		
Stroke history				0.45	0.80
Positive	4	21	11		
Negative	18	63	36		
Atrial fibrillation				16.24	<0.001
Positive	0	13	18		
Negative	22	71	29		
Renal insufficiency				3.15	0.21
Positive	0	7	6		
Negative	22	77	41		
Hypertension				2.13	0.34
Positive	16	68	33		
Negative	6	16	14		

### 3.2 Serum PGAM2 and other indicator levels in HF

The serum level of PGAM2, NT-proBNP, BNP, TNT, and Cys-C level in NYHA II, NYHA III, and NYHA IV groups are shown in Table 2. The serum PGAM2, NT-proBNP, and Cys-C in NYHA IV group were significantly higher than in NYHA II and NYHA III groups with statistical difference (*p*_all_ < 0.05); however, the serum BNP and TNT were not significantly different in the 3 groups (*p*_all_ > 0.05).

**Table 2:** The serum markers’ distribution among different grade HF subjects

Markers	NYHA II	NYHA III	NYHA IV	*F*	*p*-value
PGAM2 (pg/mL)	74.31 ± 35.76	72.01 ± 34.94	92.04 ± 51.63	3.74	0.025
NT-proBNP (pg/mL)	653.70 ± 824.40	3892.00 ± 5132.10	4692.32 ± 3446.283	3.415	0.037
BNP (ng/L)	724.90 ± 841.20	528.90 ± 736.60	821.20 ± 833.00	0.79	0.46
TNT (ng/mL)	231.30 ± 490.00	165.50 ± 505.70	299.90 ± 792.10	0.59	0.56
Cys-C (mg/L)	1.09 ± 0.30	1.41 ± 0.59	1.82 ± 0.99	5.06	0.008

### 3.3 Serum biomarkers for HF severity evaluation

**Figure 2:**
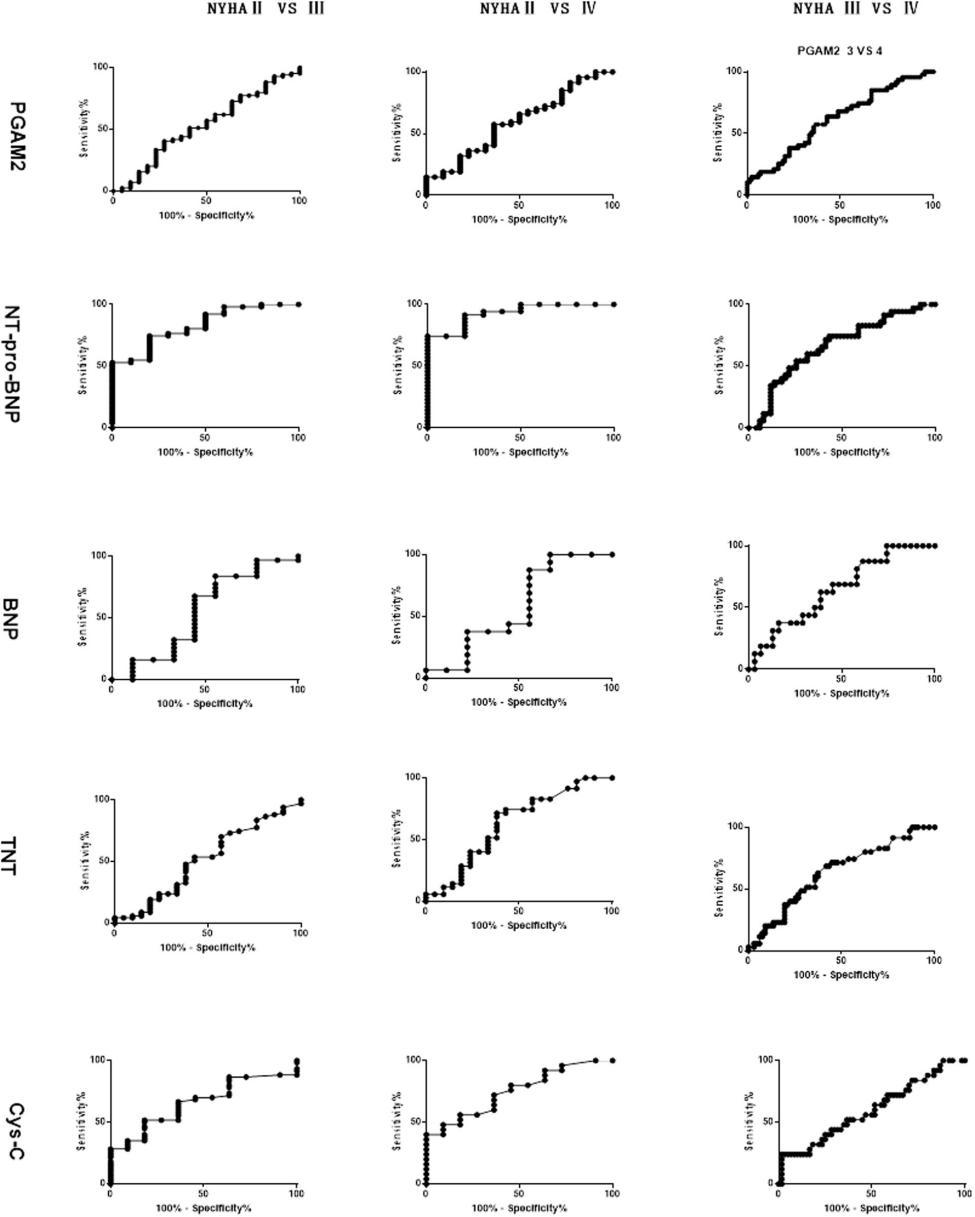
The ROC curve of serum level of PGAM2, NT-proBNP, BNP, TNT, and Cys-C in evaluation of HF severity.

The diagnostic efficacy of serum level of PGAM2, NT-proBNP, BNP, TNT, and Cys-C level in evaluation of HF severity are shown in Table 3. The diagnostic sensitivity and specificity ranged from 51.19 to 87.50% and 42.86 to 100.00%, respectively. The area under the ROC curve is demonstrated in Figure 2 with the ranges of 0.52–0.93.

**Table 3:** The diagnostic efficacy of serum level of PGAM2, NT-proBNP, BNP, TNT, and Cys-C in evaluation of HF severity

Markers	Sensitivity (%)	Specificity (%)	AUC	Likelihood ratio	Cutoff value
NYHA II vs NYHA III					
PGAM2 (pg/mL)	51.19(40.04–62.26)	54.55(32.21–75.61)	0.53(0.39–0.67)	1.13	66.69
NT-proBNP (pg/mL)	74.51(85.67)	80.00(44.39–97.48)	0.82(0.70–0.95)	3.73	866.5
BNP (ng/L)	66.74(48.63–83.32)	55.56(21.20–86.30)	0.55(0.30–0.80)	1.52	452.00
TNT (ng/mL)	70.15(57.73–80.72)	42.86(21.82–65.98)	0.52(0.37–0.67)	1.23	15.5
Cys-C (mg/L)	66.67(53.31–78.31)	63.64(30.79–89.07)	0.66(0.51–081)	1.83	1.08
PGAM2 + NT-proBNP	61.22(51.23–75.23)	68.89(33.92–90.21)	0.67(0.52–0.82)	1.81	NA
NYHA II vs NYHA IV					
PGAM2	65.96(50.69–79.14)	45.45(24.39–67.79)	0.59(0.45–0.74)	1.21	66.83
NT-proBNP (pg/mL)	74.29(56.74–87.51)	100(69.15–100)	0.93(0.85–1.00)	7.43	2493.00
BNP (ng/L)	87.50(61.65–98.45)	44.44(13.70–78.80)	0.58(0.31–0.84)	1.58	146.00
TNT (ng/mL)	71.43(53.70–85.36)	61.90(38.44–81.89)	0.63(0.47–0.79)	1.88	25.50
Cys-C (mg/L)	80.00(59.30–93.17)	54.55(23.38–83.25)	0.75(0.59–0.91)	1.76	1.02
PGAM2 + NT-proBNP	66.23(45.23–89.87)	68.87(49.56–92.11)	0.65(0.49–0.82)	1.57	NA
NYHA III vs NYHA IV					
PGAM2	63.83(48.52–77.33)	57.14(45.88–67.89)	0.61(0.52–+0.72)	1.49	71.2
NT-proBNP (pg/mL)	62.86(44.92–78.53)	60.78(46.11–74.16)	0.66(0.55–0.78)	1.60	3210.00
BNP (ng/L)	68.75(41.34–88.98)	54.84(36.03–72.68)	0.65(0.49–0.81)	1.52	280.00
TNT (ng/mL)	68.57(50.71–83.15)	56.72(44.04–68.78)	0.61(0.50–0.71)	1.59	27.5
Cys-C (mg/L)	64.00(42.52–82.03)	48.33(35.23–61.61)	0.60(0.46–0.73)	1.24	1.24
PGAM2 + NT-proBNP	62.22(41.63–80.11)	63.23(39.66–81.58)	0.64(0.49–0.79)	1.44	NA

#### 3.3.1 LVEF, LVEDD, and LA inner diameter for HF severity evaluation

**Figure 3:**
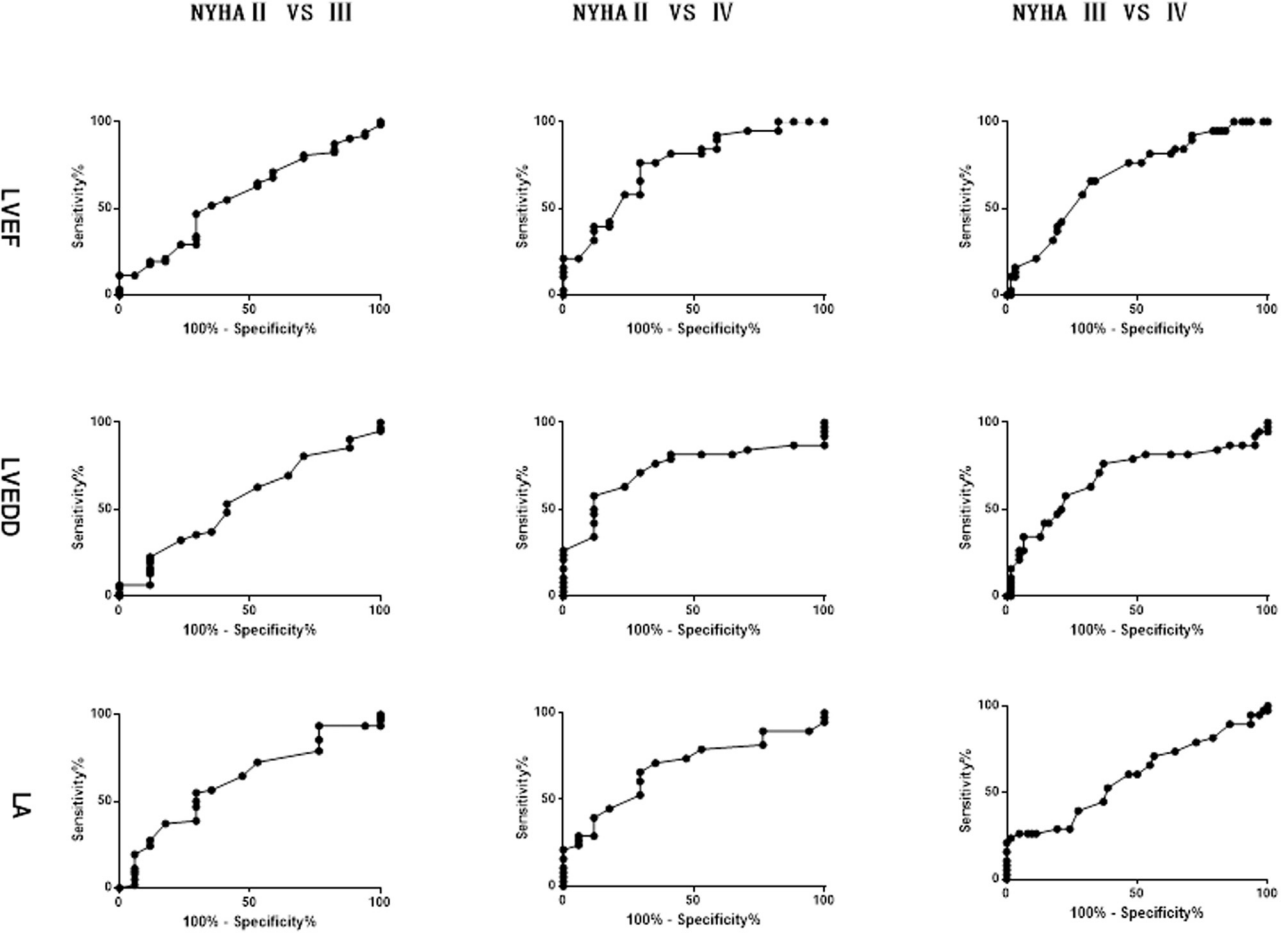
ROC curve of LVEF, LVEDD, and LA inner diameter for HF severity evaluation.

The LVEF, LVEDD, and LA inner diameter of different groups are shown in Table 4. Significant statistical difference of LVEF, LVEDD, and LA inner diameter between NYHA II, NYHA III, and NYHA IV groups were found (*p*_all_ < 0.05). The diagnostic efficacy of LVEF, LVEDD, and LA inner diameter for HF severity is demonstrated in Figure 3.

**Table 4:** Echocardiography features between different HF groups

Echocardiography	NYHA II	NYHA III	NYHA IV	*F*	*p*-value
LVEF (%)	53.41 ± 11.65	50.53 ± 12.91	41.66 ± 12.65	7.57	0.0008
LVEDD (mm)	46.18 ± 5.16	47.61 ± 7.43	53.18 ± 10.14	6.84	0.0016
LA inner diameter (mm)	36.06 ± 6.40	38.55 ± 6.76	41.67 ± 9.78	3.48	0.034

### 3.4 Survival analysis

**Figure 4:**
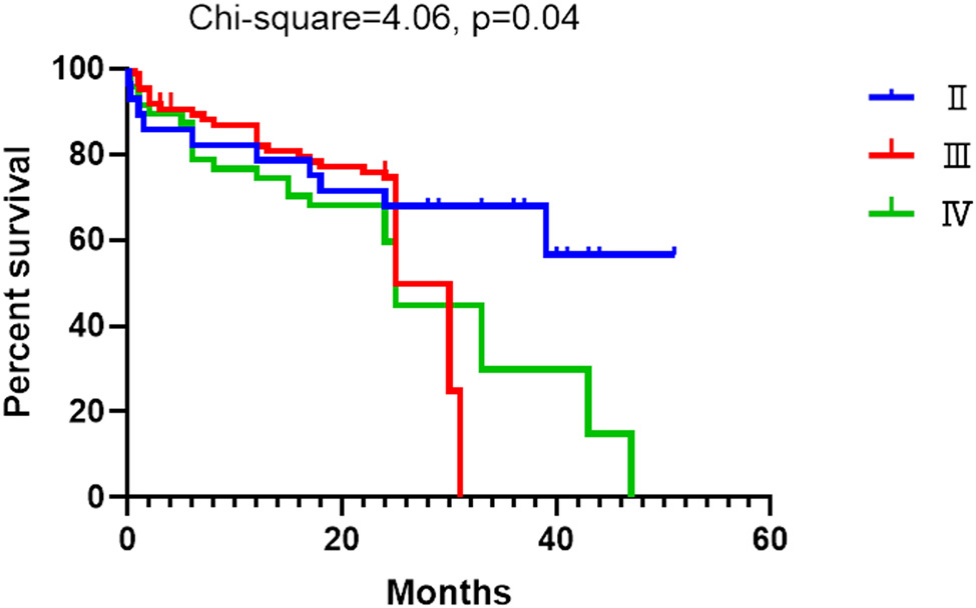
Survival curve of the HF patients divided according to the NYHA classification (*p* = 0.04 among different groups).

The overall survival among NYHA II, III, and IV groups were statistically different (*p* = 0.04) with the median survival time of 25 months for NYHA III and IV groups (Figure 4).

## 4 Discussion

Our results confirmed increased PGAM2 levels in the blood of HF patients; however, more importantly, our study reveals for the first time that PGAM2 is a good biomarker for HF diagnosis. PGAM2 was helpful in differentiating cardiac function classification (sensitivity 86% and accuracy 84%), which showed the same behavior as BNP in this respect, and was positively correlated with BNP. In addition, we also found that high levels of PGAM2 were correlated with left ventricular function.

Glucose metabolism, in particular, is thought to play an important role in the pathogenesis of HF, including changes in substrate utilization, mitochondrial dysfunction, and reduced energy transfer due to creatinine dysfunction [14,15]. Mammals have two subtypes, one is brain specific (PGAM1) and the other is muscle specific (PGAM2). Overexpression of PGAM2 in mouse embryonic fibroblasts using retrovirus vectors can promote glycolysis [16,17]. In humans, lack of the phosphoglycerate mutant enzyme leads to type X glycogen storage disease, which is characterized by exercise intolerance and cramping. Increased levels of PGAM1 protein in tissues of patients with lung cancer are associated with poor clinical prognosis. In addition, inhibiting PGAM1 protein can inhibit tumor growth [18]. A small molecule inhibitor of PGAM1 protein was developed.

The level of PGAM2 activity in cardiac tissue is the second highest, second only to skeletal muscle [19]. The expression of PGAM2 protein increased about fivefold in the canine model of HF caused by tachycardia. These results suggest that PGAM2 may be involved in the development of HF. However, the possible mechanism leading to increased PGAM2 concentration in HF patients remains unclear. PGAM2 is an enzyme in the heart’s energy metabolism [20,21]. In addition, *in vitro* studies have shown that PGAM2 by HSP90/PPAR and excessive production of reactive oxygen species to regulate myocardial glucose uptake, which could lead to change of HF myocardial metabolism as a matter of fact, free fatty acids (FFA) and glucose myocardial cells generate energy (ATP) is the main substrate opportunities and glycolysis each kind of the role of the substrate may change due to different stages of disease in advanced HF, fatty acid utilization rate dropped significantly, myocardial insulin resistance, glucose utilization rate of decline in most of the studies also show that at this stage of the disease, there is some clinical evidence [22,23] that impaired myocardial glucose and FFA utilization imbalance may lead to myocardial injury [24] in this context, increased PGAM2 appears to be a marker of disease severity and plays a role in promoting cardiac injury.

## 5 Conclusion

This study suggests that PGAM2 is a biological diagnostic approach for HF, with an accuracy comparable to that of BNP, and is a new biomarker for HF severity. There are also several limitations in the present work. First, the main limitation of our study is the small sample size. Second, the serum BNP was not statistically different among groups due to large standard deviation and small sample size, which was applied as an important biomarker for HF severity. Third, PGAM mutation status was not evaluated which may also contribute to HF. Fourth, PGAM activity is upregulated in cancers, therefore HF patients with increased PGAM should consider the risk of cancer. Nevertheless, the existing results on PGAM2’s potential as a new biomarker of HF severity are relevant and may further stimulate other relevant studies. In addition, during the establishment of the new method, the exclusion of patients with renal failure in order to avoid potential impact is a shortcoming to be further studied, and whether this method is likely to be more widely used in HF patients is to be analyzed.
